# Interpretable Machine Learning Modeling for Ischemic Stroke Outcome Prediction

**DOI:** 10.3389/fneur.2022.884693

**Published:** 2022-05-19

**Authors:** Mohamed Sobhi Jabal, Olivier Joly, David Kallmes, George Harston, Alejandro Rabinstein, Thien Huynh, Waleed Brinjikji

**Affiliations:** ^1^Department of Radiology, Mayo Clinic, Rochester, MN, United States; ^2^Brainomix Limited, Oxford, United Kingdom; ^3^Oxford University Hospitals National Health Service Trust, Oxford, United Kingdom; ^4^Department of Neurology, Mayo Clinic, Rochester, MN, United States; ^5^Department of Radiology, Mayo Clinic, Jacksonville, FL, United States

**Keywords:** ischemic stroke, artificial intelligence, machine learning, prognosis, prediction model

## Abstract

**Background and Purpose:**

Mechanical thrombectomy greatly improves stroke outcomes. Nonetheless, some patients fall short of full recovery despite good reperfusion. The purpose of this study was to develop machine learning (ML) models for the pre-interventional prediction of functional outcome at 3 months of thrombectomy in acute ischemic stroke (AIS), using clinical and auto-extractable radiological information consistently available upon first emergency evaluation.

**Materials and Methods:**

A two-center retrospective cohort of 293 patients with AIS who underwent thrombectomy was analyzed. ML models were developed to predict dichotomized modified Rankin score at 90 days (mRS-90) using clinical and imaging features, both separately and combined. Conventional and experimental imaging biomarkers were quantified using automated image-processing software from non-contract computed tomography (CT) and computed tomography angiography (CTA). Shapley Additive Explanation (SHAP) was applied for model interpretability and predictor importance analysis of the optimal model.

**Results:**

Merging clinical and imaging features returned the best results for mRS-90 prediction. The best performing classifier was Extreme Gradient Boosting (XGB) with an area under the receiver operating characteristic curve (AUC) = 84% using selected features. The most important classifying features were age, baseline National Institutes of Health Stroke Scale (NIHSS), occlusion side, degree of brain atrophy [primarily represented by cortical cerebrospinal fluid (CSF) volume and lateral ventricle volume], early ischemic core [primarily represented by e-Alberta Stroke Program Early CT Score (ASPECTS)], and collateral circulation deficit volume on CTA.

**Conclusion:**

Machine learning that is applied to quantifiable image features from CT and CTA alongside basic clinical characteristics constitutes a promising automated method in the pre-interventional prediction of stroke prognosis. Interpretable models allow for exploring which initial features contribute the most to post-thrombectomy outcome prediction overall and for each individual patient outcome.

## Introduction

Mechanical thrombectomy is currently the standard of care for patients with disabling the stroke from large vessel occlusion. Numerous trials have demonstrated its efficacy in improving survival and functional outcome for these patients (1–3). In addition, successful reperfusion does not translate into favorable recovery for a substantial proportion of patients who are treated with mechanical thrombectomy (4, 5). Thus, accurate and time-efficient risk assessment remains crucial to optimize triaging and outcomes of patients who may be candidates for stroke reperfusion therapy (6).

Several prognostication scales have been proposed to predict the functional outcome of patients with ischemic stroke (7–9). However, these scores lack the ability to fully model the complex and non-linear relationships between various prognostic factors with functional outcomes and they depend mainly on categorical rendering of clinical and few conventional imaging features.

Machine learning (ML) has emerged as a promising tool for fitting and modeling complex and multidimensional data patterns, leading to many potential applications in Medicine (10). This is owing to its ability to incorporate a large number of variables, extract nuanced information, and generalize the acquired knowledge on new unseen cases in an efficient and automatic manner, which could be particularly helpful in time-critical situations, such as acute stroke. Artificial intelligence (AI) algorithms could help to improve prediction methods by providing immediate prognostic information. Few studies have applied ML models on multimodal imaging features for modified Rankin Score (mRS) prediction in ischemic stroke, however, there still is ample room for refinement (11–13).

An important challenge for AI applications in healthcare is to overcome the confidence barrier and ensure that physicians trust the ensuing results. The black-box nature of ML algorithms makes it difficult to interpret most complex models, significant progress though has been made in the last few years in ML interpretability. One particularly promising method is Shapley Additive Explanations (SHAP), which to our knowledge has not been previously explored in depth for ML prediction of functional recovery after ischemic stroke.

The aim of this study was to develop an ML model and assess its potential in pre-interventional prediction of functional outcomes at 3 months of thrombectomy in acute ischemic stroke (AIS) using clinical and auto-extractable radiological information consistently available upon first evaluation in the emergency department. In addition, to establish an automated end-to-end system for streamlined patient triage and management decision support in stroke.

## Materials and Methods

### Dataset

The study included 443 patients from two academic centers with a confirmed diagnosis of AIS, due to large vessel occlusion in the anterior circulation [internal carotid or middle cerebral artery (MCA)] confirmed on computed tomography angiography (CTA) who underwent mechanical thrombectomy between 2014 and 2020. All included patients underwent standardized acute stroke imaging that includes non-contrast head CT and CTA of the head and neck. Patients without CTA or with any missing pertinent clinical or radiological data were excluded. The primary clinical outcome of interest was the modified Rankin score at 90 days (mRS-90). The study protocol was approved by the Institutional Review Board. The data that support the findings of this study could be available from the corresponding author upon reasonable request.

### Feature Extraction

Collected clinical and demographic characteristics were age, sex, baseline National Institutes of Health (NIH) Stroke Scale (NIHSS), time from symptom onset to admission, and comorbidities (diabetes, hypertension, hyperlipidemia, previous stroke, and cardiovascular disease, such as myocardial infarction and arrhythmia), in addition to blood glucose and blood pressure levels. Interventional and post-interventional features, such as modified treatment in cerebral infarction (mTICI) score informing reperfusion status, were excluded from the models in line with the main purpose of the study.

Quantitative imaging feature extraction was performed using e-Stroke software (Brainomix, Oxford, UK) for automated calculation of Alberta Stroke Program Early CT Score (ASPECTS; e-ASPECTS) and estimated acute infarct volumes on non-contrast CT (14–17). e-ASPECTS uses ML classification to distinguish and segment regions that contain signs consistent with the acute ischemic change in order to output both (total and per ASPECTS region) and total e-ASPECTS volumes. Additional novel features that were extracted using e-Stroke software included non-acute infarct volume, total brain volume, and atrophy, which were quantified using cerebrospinal fluid (CSF) segmentation volumes in both the lateral ventricles and the cortical sulci separately and expressed as percentages. e-CTA (Brainomix, Oxford, UK) identifies large vessel occlusion site and quantifies the volume of collateral circulation deficit both as a percentage of the total volume and using the CTA collateral score (CTA-CS) (18–20). Novel experimental outputs from e-CTA included the absolute volume of the vessel density deficit in MCA territory relative to the contralateral hemisphere.

### Feature Pre-processing

Baseline features were categorized into clinical and imaging feature groups. Standardization scaling of continuous and ordinal feature values was applied to obtain a mean of zero and a standard deviation (SD) of 1, in order to facilitate the algorithm learning process and improve the prediction results. Random splitting of the datasets into a training set and a testing set was applied with a ratio of 75–25%, respectively. The mRS-90 was dichotomized with mRS 0–2 representing a good functional outcome.

The features were divided into four subsets, which are as follows: (1) clinical features, (2) imaging features, (3) combined clinical and imaging features, and (4) selected features. A model-based approach was applied using sequential backward feature selection with a bagging classifier, where an algorithm sequentially removes features from the full feature set until the removal of further features decreases the classifier performance.

### Statistical Analysis

Statistical assessment of each clinical and image-based feature in relation to mRS-90 was assessed using the chi-square test for the categorical variables, Wilcoxon Rank-Sum test, and *t*-test for the ordinal and continuous variables depending on the normality of their distributions. Statistical analysis was done using Python (version 3.9) and the SciPy library. Values of *p* < 0.05 were considered statistically significant.

### ML Model Development and Testing

For the purpose of mRS-90 prediction, supervised ML classification methods were deployed. The ML algorithms used were as follows: k-nearest neighbors, random forests (RF), gradient boosting (GB), and Extreme Gradient Boosting (XGBoost). The models were constructed using the Scikit-learn library.

As a first step, *k*-fold cross-validation of 10-folds was performed during the training for each model, which divides the training set into 10 subsets (9 for training and 1 for validation), where the training and validation sets change and iterate over the 10-folds. The model hyperparameters were optimized by means of a grid search approach, where for every model and for each hyperparameter a set of possible values was manually defined and evaluated exhaustively in every iteration to determine the values corresponding to the model's highest performance, with an area under the receiver operating characteristic curve (AUC) as scoring metric. The ML models were trained using each of the 4 different feature categories. Subsequently, for every feature group, we tested the models' performance on the testing set of patients.

From the output of the grid search, the best performing model was chosen. Finally, automatic Bayesian hyperparameter tuning with the Optuna framework was used on the best performing model to boost its performance and achieve finer tuning. The evaluation metrics used were accuracy, F1 score (for mRS-90 ≤ 2 and mRS-90 > 2 predictions), and AUC.

To enhance the model's explainability and perform a feature importance analysis, we used the method SHAP, which is based on game theory and consists of computing Shapley values reflecting the contribution of each feature in the predictions of the model (21–23). The method allows for the identification of features with the most influence on model output and measures the impact if each variable was to be removed while taking into account the interaction with other variables that provide insight on the relative importance of the features used by the model for its prediction decision process.

**Figure 1:**
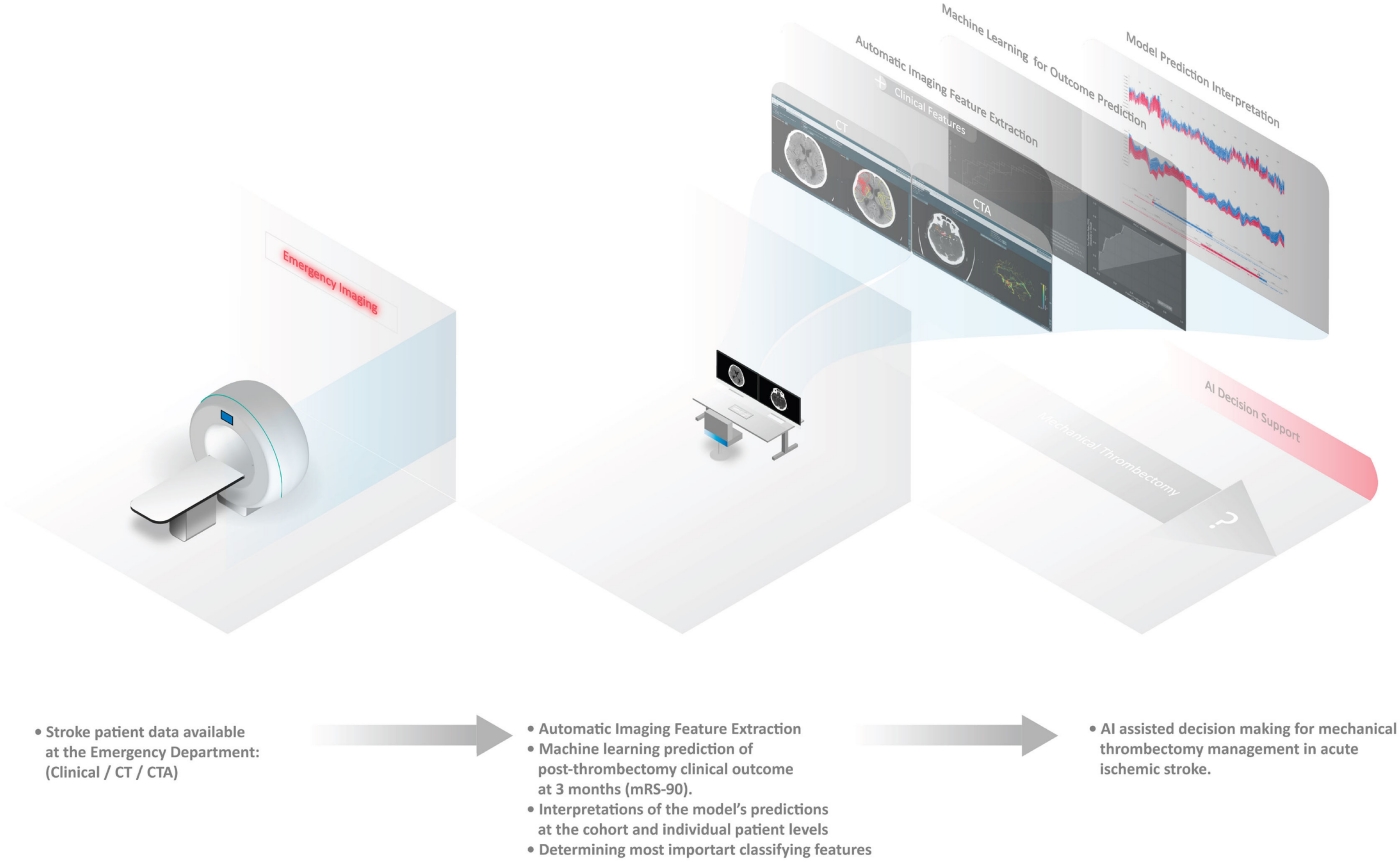
Automated pipeline system for stroke functional outcome prediction at the emergency imaging providing artificial intelligence (AI) decision support for mechanical thrombectomy.

An illustrative summary of the methods is provided in Figure 1.

## Results

### Patient Population

Of 443 total patients (266 from the first center and 177 from the second center), 293 patients met the study inclusion criteria. The remainder of the patients were excluded for lacking relevant variables mainly CTA images. In total, 101 patients had a favorable functional outcome (mRS-90 ≤ 2), while 192 patients had unfavorable functional outcomes (mRS-90 > 2). The median age of included patients was 71 years and 49% (*n* = 143) were women (Table 1).

**Table 1:** Statistical feature comparison between the two outcome groups.

**Features**	**mRS-90 ≤ 2 (*n* = 101)**	**mRS-90 > 2 (*n* = 192)**	***P*-value**
**Clinical features**
Age, median (IQR)	63 (51–74)	75 (63–84)	<0.0001
Sex			0.016
Female, *n* (%)	39 (39%)	104 (54.2%)	
Male, *n* (%)	62 (61%)	88 (45.8%)	
NIHSS score, median (IQR)	13 (7–18)	18 (13–22)	<0.0001
Time to admission, median (IQR)	107 (68–186)	135 (68–302)	0.107
**Imaging features**
Occlusion side			0.017
Right, *n* (%)	57 (56%)	79 (41%)	
Left, *n* (%)	44 (44%)	113 (59%)	
Occlusion location			0.280
ICA Terminus, *n* (%)	25 (25%)	55 (29%)	
M1, *n* (%)	50 (49%)	99 (51%)	
M2, *n* (%)	25 (25%)	34 (18%)	
M3, *n* (%)	1 (1%)	4 (2%)	
e-ASPECTS, median (IQR)	9 (8–10)	9 (7–10)	0.002
Acute ischemic Volume (mL), median (IQR)	9.14 (5–20)	12.52 (5–28)	0.047
Non-acute ischemic volume (mL), median (IQR)	0.39 (0–0)	0.50 (0–1)	0.040
**Local acute ischemic volume**
M1 (mL), median (range)	0.0 (0.0–9.5)	0.0 (0.0–13.7)	0.006
M2 (mL), median (range)	0.3 (0.0–8.8)	0.8 (0.0–18.8)	0.005
M3 (mL), median (range)	0.0 (0.0–12.5)	0.4 (0.0–20.2)	0.002
M4 (mL), median (range)	0.0 (0.0–7.5)	0.0 (0.0–11.7)	0.004
M5 (mL), median (range)	0.8 (0.0–17.9)	1.3 (0.0–32.0)	0.034
M6 (mL), median (range)	0.1 (0.0–23.0)	0.8 (0.0–25.3)	0.012
Caudate (mL), median (range)	0.0 (0.0–2.6)	0.0 (0.0–2.6)	0.540
Insula (mL), median (range)	0.0 (0.0–8.0)	4.7 (0.0–8.0)	0.201
Internal capsule (mL), median (range)	0.0 (0.0–4.7)	0.0 (0.0–4.8)	0.744
Lentiform (mL), median (range)	2.3 (0.0–5.8)	2.6 (0.0–5.8)	0.679
Brain volume (L), mean (±SD)	1.30 (±0.16)	1.26 (±0.15)	0.043
Cortical CSF volume (%), median (IQR)	6.16 (4–9)	8.7 (6–10)	<0.0001
Lateral ventricle volume (%), median (IQR)	2.4 (1–3)	3.4 (2–5)	<0.0001
Circulation deficit volume, median (IQR)	15.8 (1–36)	30.42 (6–54)	0.001
CTA CS score, median (IQR)	3.0 (2–3)	2.0 (1–3)	<0.001

### Univariate Statistical Analysis

Favorable clinical outcome was significantly associated with younger age (*p* < 0.0001), female sex (*p* = 0.016), and lower baseline NIHSS score (*p* < 0.0001). Patient comorbidities were not significantly different between outcome groups (Supplementary Table S1) and therefore were not included in the development of ML models.

Non-contrast CT imaging features associated with favorable outcomes included greater e-ASPECTS (*p* = 0.002), larger brain volume (*p* = 0.043), smaller cortical CSF volume (*p* < 0.0001), smaller lateral ventricle volume (*p* < 0.0001), smaller acute ischemic volume (*p* = 0.047), and non-acute ischemic volume (*p* = 0.040). Collateral circulation deficit volume on CTA was significantly lower in the favorable outcome group (*p* = 0.001; Table 1).

### ML Model

Performance evaluation of each ML model following grid search optimization is presented in Supplementary Table S2. The selected features were as follows: baseline NIHSS, age, occlusion side, local M5 infarct volume, local lentiform infarct volume, brain volume, percentage of lateral ventricle volume, collateral vessel deficit volume, and the time interval from symptoms onset to admission.

**Figure 2:**
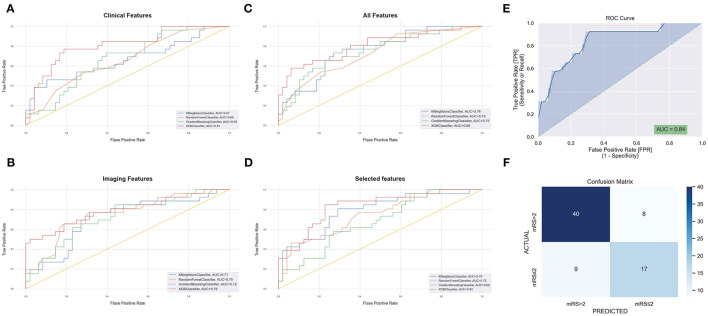
Receiver operating characteristic curves (ROCs) with areas under curves for modified Rankin score at 90 days (mRS-90) prediction after grid-search optimization using baseline clinical features **(A)**, imaging features **(B)**, all features **(C)**, and selected features **(D)**, the orange-dashed line represents random guessing with an area under the receiver operating characteristic curve (AUC) of 0.5. The AUC **(E)** and confusion matrix **(F)** of the best performing model following Bayesian hyperparameter tuning using the selected features.

We calculated accuracy and AUC for each of the feature groups on the training data set. For clinical data, the model with the highest AUC score was the XGBoost classifier (XGB) with an AUC of 81%. For imaging features, the best model was XGB at 79% AUC. For combined clinical and imaging features, the best model was also XGB with an AUC of 80%. Selected features yielded an AUC of 84% (Figures 2A–D).

The XGB model was selected for further optimization as it consistently achieved a high performance in the four feature groups and had the highest overall AUC scores. Using Bayesian hyperparameter tuning with a stratified cross-validation of 10-folds to refine the XGB model, the final performance metrics on the testing set of patients were AUC = 84%, accuracy = 77%, F1-score (mRS ≤ 2) = 67%, and F1-score (mRS > 2) = 82% for the selected features. The final results are shown in Figures 2E,F.

**Figure 3:**
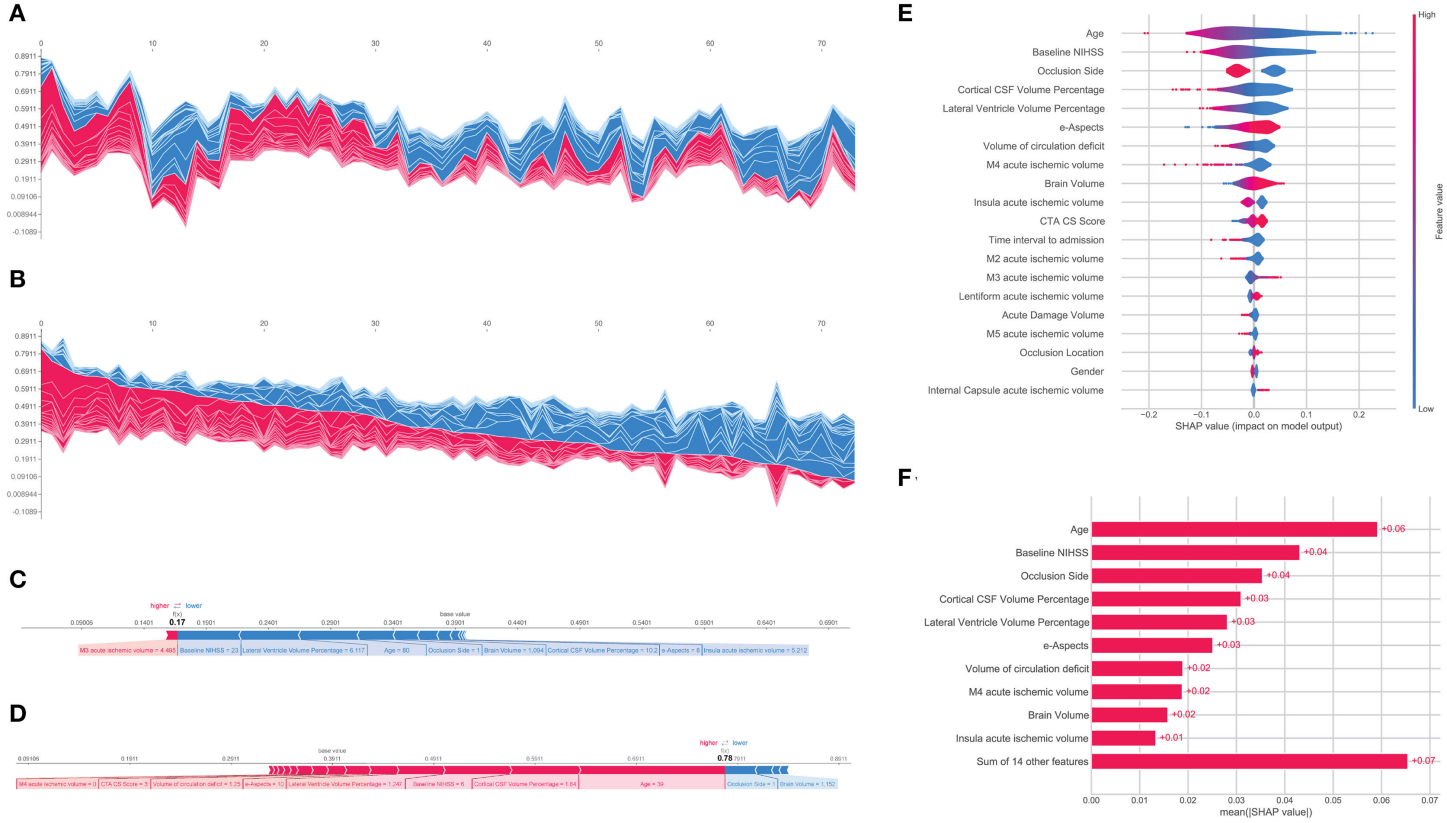
Shapley Additive Explanation (SHAP) force plot of the testing set with the vertical axis representing model outcome and the horizontal axis representing the testing population sample ordered by feature similarity **(A)** and by model output **(B)**. Examples of the model output for an individual patient with the determining feature values that influenced the classification decision from the poor outcome group **(C)** and the favorable outcome group **(D)**. SHAP summary plot showing the distribution of each patient feature and how it affects the model outcome through its SHAP value **(E)**. Absolute mean SHAP values for the global effect of every feature effect on the model output **(F)**.

Following prediction modeling of mRS-90, a feature importance rank for the patient cohort was established by calculating SHAP values for XGBoost which revealed that the top indicators of clinical outcome prediction for the model were by order of importance, i.e., age, baseline NIHSS, occlusion side, cortical CSF volume percentage, lateral ventricle volume percentage, e-ASPECTS, and circulation deficit volume (Figure 3E). The overall impact of each feature is represented by feature SHAP general values as shown in Figure 3F and model predictions were able to be reviewed and assessed regarding each predictor for each patient instance. The SHAP force plot allows for an interactive visualization of all the study populations clustered by their feature value similarity and ranging according to their specific model output (Figures 3A,B). Individual patient predictions can be extracted to visualize which features played a role in their classification and what their feature values were. Examples of predictions for a patient with poor outcomes and a patient with favorable outcomes are shown in Figures 3C,D, respectively.

## Discussion

In this study, we have developed and tested ML models to predict the 3-month functional outcome of patients with AIS and large vessel occlusion treated with mechanical thrombectomy using only clinical and imaging features available in the emergency department. Employing very simple baseline clinical information and automatically extracting quantitative imaging features from the baseline CT and CTA, our final model achieved very good predictive accuracy. Some of the features incorporated into our predictive model had not been previously examined, such as radiological markers of brain atrophy (brain volume, cortical CSF volume, and ventricular volume). In addition, we presented the feasibility of building interpretable ML models for stroke outcome prediction. The reporting of our prediction model includes information on what features weighed more heavily on the prediction that the algorithm utilized to construct the model.

The high evaluation metrics results in our study could be attributed to the newly introduced quantifiable features from automated image post-processing technology and the use of Bayesian hyperparameter tuning. Although all the included features contributed to the model performance, the most important features for the final model outcome prediction were as follows: age, baseline NIHSS, occlusion side, degree of brain atrophy (primarily represented by cortical CSF volume and lateral ventricle volume), early ischemic core (primarily represented by e-ASPECTS), and circulation deficit volume on CTA. This demonstrates the opportunity for multiple automatic imaging biomarkers extractable from routinely acquired imaging modalities (CT and CTA) to improve the precision of patient profiling for AIS management.

The complexity of ML models leads to challenges in defining the reasoning behind their predictions, thus potentially hampering clinical adoption. In this study, the SHAP methodology provided explicability to the ML model at the cohort level and for each individual patient prediction with user-friendly visualization tools for demonstration purposes. These types of approaches have the potential for resolving the “trust barrier” between clinicians and AI algorithms and could help to increase clinical engagement with ML as future practice tools.

Our work results are consistent with and validate findings from previous studies, which have evaluated ML models for outcome prediction after AIS. Jiang et al. illustrated that ML applied to clinical and advanced imaging features had superior performance in binary mRS-90 prediction when compared to the Stroke Prognostication using Age and NIHSS (SPAN-100) scale (12). They reported the best model AUC of 80% using the 6 best performing features that include CT perfusion features (baseline NIHSS, age, glucose at admission, ischemic core volume on CT perfusion, penumbra volume on CT perfusion, and CTA-clot burden score) (12). Brugnara et al. reported a model for predicting mRS-90 after endovascular treatment for AIS that achieved an AUC of 74% using just baseline clinical and radiological features (13). The incorporation of features from CT perfusion did not improve the predictive performance of their model, but the inclusion of angiographic and post-interventional features significantly improved the predictive performance with an AUC of 85%. The most important parameters for their mRS-90 prediction were NIHSS after 24 h, pre-morbid mRS, and volume of final infarction volume on post-interventional CT (13).

During model development, we experimented with incorporating features related to the endovascular intervention and post-interventional clinical and radiological features, such as TICI score, which as expected did increase the performance of the predictive models. However, the goal of the study was not just to merely develop a prognostic tool or achieve the highest prediction metrics possible but also to explore how the potential of ML models for decision supports in the setting of initial screening at the emergency department prior to the intervention and to identify the patients who would highly benefit if a mechanical thrombectomy procedure was to be performed. For that purpose, we intentionally chose to restrict the features to only those readily available at first scan and evaluation upon urgent patient arrival and excluded the post-interventional features from our final analysis. This interpretable approach could have promising applications and provide helpful service for stratifying patients with large vessel occlusion stroke prior to the endovascular procedure, leading to enhanced acute management decision-making.

### Limitations

Limitations to our study may relate to the population size, which is although relatively large as a two-center study, similar to most ML studies, it could benefit from a larger cohort size for ML purposes. Datasets with diverse origins and a higher number of participants are warranted to further validate the robustness of the models for future generalizability on independent cohorts. In addition, with the absence of consistent information on pre-morbid functional status, we have not included this variable. Future planned steps exist for validating these tools prospectively and on larger multi-center datasets for further optimization of this approach.

## Conclusion

Automated approaches could help to streamline and inform the decision-making process prior to thrombectomy in AIS at the emergency department. Our study highlights the value and accuracy of ML approaches integrating basic clinical information and automated imaging features in the pre-interventional prediction of functional outcomes 3 months from mechanical thrombectomy and the role of AI in both extracting useful information from routine imaging and individualizing prognostication and management decision-support systems in AIS. Progress made in ML interpretability is paving the way for more transparent modeling, which is becoming essential in the medical realm and for identifying important new predictors of stroke outcome.

## Data Availability Statement

The raw data supporting the conclusions of this article will be made available by the authors, without undue reservation.

## Ethics Statement

The studies involving human participants were reviewed and approved by the Mayo Clinic Institutional Review Board (IRB). Written informed consent for participation was not required for this study in accordance with the national legislation and the institutional requirements.

## Author Contributions

WB and DK contributed to conception and design of the study. GH and OJ provided software support for image post-processing. MJ performed the machine learning analysis, interpretation, visualization, and manuscript draft redaction. WB, DK, GH, AR, and TH supervised and contributed to manuscript revision, editing, and approval of the submitted version. All authors contributed to the article and approved the submitted version.

## Conflict of Interest

GH and OJ receive salary support and share options from Brainomix Ltd. The remaining authors declare that the research was conducted in the absence of any commercial or financial relationships that could be construed as a potential conflict of interest.

## Publisher's Note

All claims expressed in this article are solely those of the authors and do not necessarily represent those of their affiliated organizations, or those of the publisher, the editors and the reviewers. Any product that may be evaluated in this article, or claim that may be made by its manufacturer, is not guaranteed or endorsed by the publisher.

## Abbreviations

AIS: Acute Ischemic Stroke
ASPECTS: Alberta Stroke Program Early CT Score
AUC: Area Under the Receiver Operating Characteristic Curve
AI: Artificial Intelligence
CSF: Cerebrospinal Fluid
CT: Computed Tomography
CTA: Computed Tomography Angiography
CTA-CS: CTA Collateral Score
XGB: Extreme Gradient Boosting
GB: Gradient Boosting
IQR: Interquartile Range
KNN: K Nearest Neighbor
ML: Machine Learning
mRS: modified Rankin Score
mRS-90: modified Rankin Score At 90 Days
NIHSS: National Institutes of Health Stroke Scale
RF: Random Forests
SHAP: Shapley Additive Explanations
TICI: Treatment in cerebral infarction.
