# Supplementary material for: Interpretable Machine Learning Modeling for Ischemic Stroke Outcome Prediction
Source: Front Neurol. 2022 May 19;13:884693. doi: 10.3389/fneur.2022.884693 (PMC9160988; doi:10.3389/fneur.2022.884693)
Supplement: Supplementary file 1 [file Table_1.DOCX]

**Online Supplement**

**Supplementary Table 1.** Clinical and comorbidity features from one center.

| **Features** | **mRS-90 ≤ 2**  **(n=76)** | **mRS-90 > 2**  **(n=115)** | ***P* value** |
| --- | --- | --- | --- |
| Clinical features |  |  |  |
| Age, median (IQR) | 62 (49-73) | 74 (63-81) | <0.001 |
| Sex |  |  | 0.091 |
| Female, n (%) | 30 (40%) | 61 (53%) |  |
| Male, n (%) | 46 (60%) | 54 (47%) |  |
| NIHSS score, median (IQR) | 12 (7-18) | 19 (14-22) | <0.001 |
| Past stroke, n (%) | 12 (16%) | 18 (16%) | 0.859 |
| Time to admission, median (IQR) | 108 (70-180) | 138 (61-272) | 0.041 |
| Hypertension, n (%) | 33 (43%) | 66 (57%) | 0.081 |
| Systolic BP, median (IQR) | 144 (129-160) | 148 (127-163) | 0.680 |
| Diastolic BP, median (IQR) | 80 (69-93) | 82 (68-96) | 0.297 |
| Hyperlipidemia, n (%) | 22 (29%) | 45 (39%) | 0.197 |
| Cardiovascular comorbidities, n (%) | 42 (55%) | 52 (45%) | 0.225 |
| Diabetes, n (%) | 8 (10%) | 21 (18%) | 0.210 |
| Glycemia, median (IQR) | 116 (103-131) | 118 (105-145) | 0.209 |

**Supplementary Table 2.** Evaluation metrics of the initial machine learning models.

| **Model** | **Clinical features** | | | | **Imaging features** | | | | **All features** | | | | **Selected Features** | | | |
| --- | --- | --- | --- | --- | --- | --- | --- | --- | --- | --- | --- | --- | --- | --- | --- | --- |
|  |  | **ACC** | **AUC** |  | | **ACC** | **AUC** |  | | **ACC** | **AUC** |  | | **ACC** | **AUC** |  |
| **KNN** |  | 69% | 67% |  | | 69% | 71% |  | | 70% | 76% |  | | 74% | 79% |  |
| **RF** |  | 68% | 66% |  | | 70% | 75% |  | | 72% | 73% |  | | 72% | 76% |  |
| **GB** |  | 64% | 68% |  | | 68% | 72% |  | | 69% | 75% |  | | 62% | 68% |  |
| **XGB** |  | 74% | 81% |  | | 76% | 79% |  | | 80% | 80% |  | | 74% | 83% |  |

KNN: K Nearest Neighbor, RF: Random Forest, GB: Gradient Boosting. XGB: Extreme Gradient Boosting. AUC: Area Under the Curve. ACC: Accuracy.


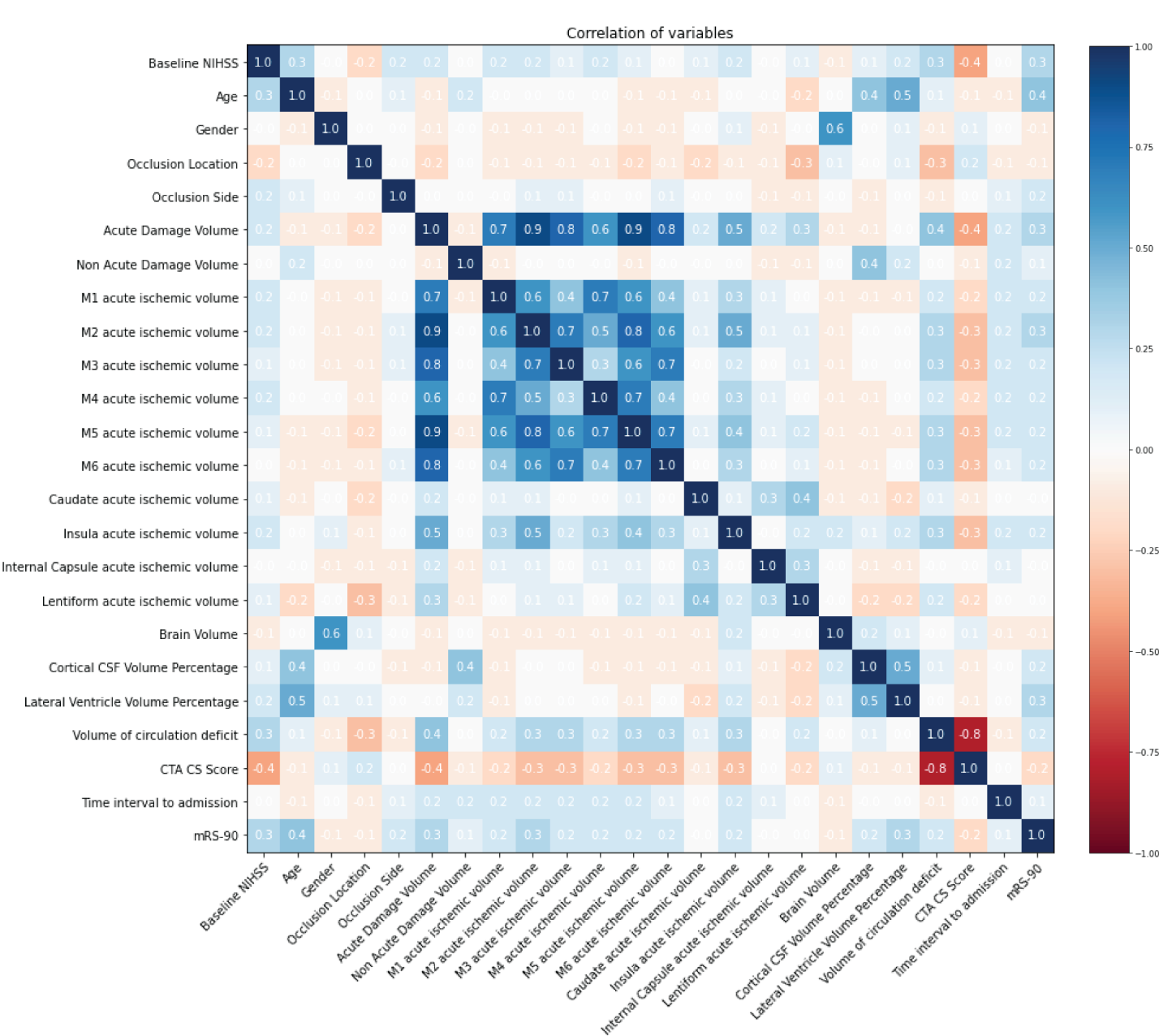


**Supplementary Figure 1.** Correlation heatmap matrix of Pearson coefficients between features and outcome.
